# Dental pulp stem cells as a therapy for congenital entero-neuropathy

**DOI:** 10.1038/s41598-022-10077-3

**Published:** 2022-04-28

**Authors:** Koichiro Yoshimaru, Takayoshi Yamaza, Shunichi Kajioka, Soichiro Sonoda, Yusuke Yanagi, Toshiharu Matsuura, Junko Yoshizumi, Yoshinao Oda, Naoko Iwata, Chiho Takai, Shinsuke Nakayama, Tomoaki Taguchi

**Affiliations:** 1 Department of Pediatric Surgery, Kyushu University Graduate School of Medical Sciences, Fukuoka, Japan; 2 Department of Molecular Cell Biology and Oral Anatomy, Kyushu University Graduate School of Dental Science, 3-1-1 Maidashi, Higashi-ku, Fukuoka, 812-8582 Japan; 3 Department of Clinical Pharmacology, Kyushu University Graduate School of Medicine, 3-1-1 Maidashi, Higashi-ku, Fukuoka, 812-8582 Japan; 4 Department of Oral and Maxillofacial Surgery, Fukuoka Dental College, Fukuoka, Japan; 5 Department of Anatomic Pathology, Kyushu University Graduate School of Medical Sciences, Fukuoka, Japan; 6 Department of Cell Physiology, Nagoya University Graduate School of Medicine, 65 Tsurumai-cho, Showa-ku, Nagoya, 466-8550 Japan; 7 Present Address: Department of Pharmaceutical Sciences, International University of Health and Welfare, 137-1 Enokizu, Okawa, Fukuoka 831-8501 Japan

**Keywords:** Physiology, Gastroenterology, Medical research

## Abstract

Hirschsprung’s disease is a congenital entero-neuropathy that causes chronic constipation and intestinal obstruction. New treatments for entero-neuropathy are needed because current surgical strategies have limitations5. Entero-neuropathy results from enteric nervous system dysfunction due to incomplete colonization of the distal intestine by neural crest-derived cells. Impaired cooperation between the enteric nervous system and intestinal pacemaker cells may also contribute to entero-neuropathy. Stem cell therapy to repair these multiple defects represents a novel treatment approach. Dental pulp stem cells derived from deciduous teeth (dDPSCs) are multipotent cranial neural crest-derived cells, but it remains unknown whether dDPSCs have potential as a new therapy for entero-neuropathy. Here we show that intravenous transplantation of dDPSCs into the Japanese Fancy-1 mouse, an established model of hypoganglionosis and entero-neuropathy, improves large intestinal structure and function and prolongs survival. Intravenously injected dDPSCs migrate to affected regions of the intestine through interactions between stromal cell-derived factor-1α and C-X-C chemokine receptor type-4. Transplanted dDPSCs differentiate into both pacemaker cells and enteric neurons in the proximal colon to improve electrical and peristaltic activity, in addition to their paracrine effects. Our findings indicate that transplanted dDPSCs can differentiate into different cell types to correct entero-neuropathy-associated defects.

## Introduction

Hirschsprung’s disease (HSCR) and its allied disorders are congenital entero-neuropathies that occur with an incidence of ~ 1 per 5000 livebirths in Japan^1–3^. HSCR is a potentially fatal disorder with clinical features that include constipation, abdominal distension, megacolon and intestinal obstruction^1–3^. The current management of HSCR involves surgery to remove or bypass affected segments of the bowel^4^. Nevertheless, many children suffer from life-long complications due to the dysfunction of ‘healthy’ bowel retained during surgery^5^ and the consequences of additional palliative surgery such as proctectomy and enterostomy. Therefore, novel therapies are urgently needed to improve outcomes and quality of life in patients with HSCR.

HSCR was first reported in 1888 by Hirschsprung, who described two infants with local defects of enteric neurons^6^. The pathogenesis of HSCR is incompletely understood but thought to involve the failure of enteric neural crest-derived cells (NCCs) to complete their colonization of the distal intestine during foetal development. Since the initial discovery that enteric neural circuits play an essential role in peristaltic movements of the gut (the ‘law of the intestine’ proposed by Bayliss and Starling in 1899)^7^, it has become established that intestinal motility is coordinated by an intrinsic nervous system. In the 1990s, the interstitial cells of Cajal (ICCs) were identified as specialized cells that act as an intestinal pacemaker^8^. ICCs (especially those in the myenteric plexus) are thought to contribute to the spatial organization of gut motility because of their network-like structure. Cooperation between the enteric nervous system (ENS) and ICC ‘pacemaker’ system is essential for the coordination of complex intestinal movements^9,10^, and this cooperation between the ENS and ICCs is thought to be impaired in HSCR and its allied disorders. The complex interaction between the ENS and ICCs may explain why familial *RET* mutations, which are known to affect colonization of the intestine by NCCs, exhibit incomplete penetrance^11^.

Dental pulp stem cells derived from deciduous teeth (dDPSCs) are cranial neural crest-derived mesenchymal stem cells (MSCs) that can differentiate to form structures such as the pulp-dentin complex due to their multilineage potential^12–14^. Our cultured dDPSCs are multipotent cells that express NCC markers and have low levels of class II HLA, which is important for immune tolerance^15^. In view of their characteristics, we hypothesized that dDPSCs may have potential for development into a novel cell-based therapy for entero-neuropathies. Here we report the effects of intravenous transplantation of dDPSCs into the Japanese Fancy-1 (JF1) mouse, which is an established animal model of entero-neuropathy. The JF1 mouse carries a homozygous mutation at the piebald locus (*Ednrb*^*s*^) that encodes the endothelin-B receptor (ETBR), and the phenotype is characterized by a sparse network of myenteric ganglia, especially in the proximal colon^16–19^. We show that intravenously injected dDPSCs migrate to affected regions of the intestine through interactions between stromal cell-derived factor-1α (SDF1α) and C-X-C chemokine receptor type-4 (CXCR4). Furthermore, the transplanted dDPSCs differentiate into both pacemaker cells and enteric neurons in the proximal colon to restore the slow rhythmic potentials observed in wild-type mice. Our findings imply that congenital entero-neuropathies involve defects of the intestinal pacemaker system and that dDPSCs have potential for development into a new treatment for HSCR in humans.

## Results

### dDPSCs migrate to the intestine

**Figure 1:**
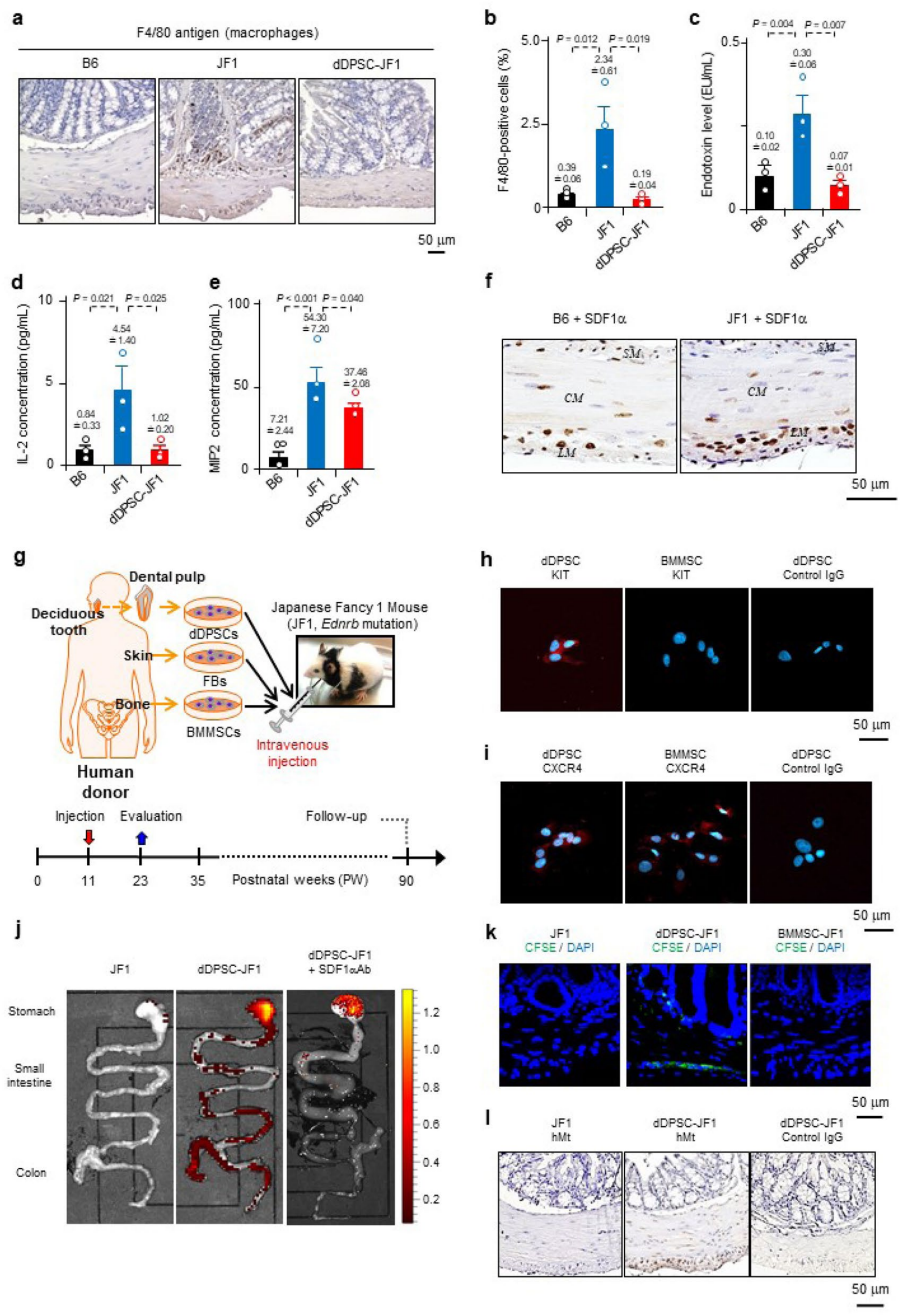
Intestinal migration of dDPSCs. **(a)** Macrophages in the proximal colon. (**b**) Macrophage percentage in the proximal colon (mean ± SEM, *n* = 3). (**c–e**) Intestinal endotoxin, IL-2 and MIP2 levels (mean ± SEM, *n* = 3). (**f**) Proximal colon SDF1α expression (× 200). CM: circular muscle; LM: longitudinal muscle; SM: submucosal layer. (**g**) Experimental schedule. (**h**,**i**) Stem cell KIT and CXCR4 expression. **j**, Mouse intestine (ex vivo fluorescence imaging; *n* = 1). (**k**) CFSE-labelled cells 3 days post-transplantation (proximal colon, × 200). Representative of ≥ 3 experiments. (**l**) hMt-containing cells 12 weeks post-transplantation (proximal colon, × 200). (**a,f,h,i,k,l**) Representative of ≥ 2 independent experiments.

JF1 mice had a shorter body length than age-matched wild-type B6 mice, and their colons exhibited faecal stasis and reduced mucosal thickness (Extended Data Fig. 1a–c). Proximal colon levels of endothelin receptor type B (*Ednrb*) mRNA and protein were lower in JF1 mice than in B6 mice (Extended Data Fig. 1d–f). Compared with B6 mouse colon, JF1 mouse colon had higher levels of macrophage infiltration, intestinal endotoxin, interleukin-2 (IL-2) and macrophage inflammatory protein-2 (MIP2) (Fig. 1a–e), higher faecal bacterial content, a higher proportion of Bacteroidetes (but not *Firmicutes* or other bacteria) in the intestinal microbiota, and a lower Gini-Simpson index (Extended Data Fig. 2). Hence, JF1 mouse colon exhibits intestinal inflammation and bacterial imbalance similar to that observed in *Ednrb* knock-out mice^20^.

Stromal cell-derived factor-1α (SDF1α) is upregulated in inflamed tissue, and its receptor (CXCR4)^21^ is expressed in MSCs such as dDPSCs and bone marrow MSCs (BMMSCs). SDF1α-positive cells were abundant in the myenteric plexus of JF1 mouse proximal colon (Fig. 1f), raising the possibility that dDPSCs and BMMSCs might target intestinal regions exhibiting hypoganglionosis-related local inflammatory changes. Therefore, our subsequent experiments investigated the effects of intravenous injection of dDPSCs into JF1 mice at postnatal week 11 (P11W), and in some experiments we compared the effects of transplanted dDPSCs with those of intravenously injected BMMSCs or human skin fibroblasts (FBs) (Fig. 1g). Cultured dDPSCs at passage 3 (P3) expressed KIT (CD117, a receptor tyrosine kinase) and CXCR4 (Fig. 1h,i), retained the characteristics of neural crest cells, and had low immunogenicity (Extended Data Fig. 3).

We investigated the migration of transplanted dDPSCs (P11W) using cells labelled with 1,1'-dioctadecyl-3,3,3',3'-tetramethylindotricarbocyanine iodide (DiR). In vivo fluorescence imaging of dDPSC-transplanted JF1 mice (dDPSC-JF1 mice) revealed that DiR-labelled dDPSCs were widely distributed on day 1 but had migrated to the intestine by day 7 (Extended Data Fig. 4a). Ex vivo fluorescence imaging 9 weeks after transplantation revealed a high fluorescence intensity in the intestine (stomach, distal ileum and proximal colon), spleen and liver (Fig. 1j and Extended Data Fig. 4b). Intraperitoneal administration of a neutralizing antibody to SDF1α almost completely abolished the intestinal accumulation of DIR-labelled dDPSCs (Fig. 1j and Extended Data Fig. 4a,b), suggesting that SDF1α-CXCR4 interactions play a role in dDPSC migration to the intestine. Little or no fluorescence was detected in B6 mice injected with DiR-labelled dDPSCs (Extended Data Fig. 4c), implying that dDPSCs migrate specifically to regions of hypoganglionosis. Carboxyfluorescein diacetate succinimidyl ester (CFSE)-labelled dDPSCs were observed in the proximal colon of dDPSC-JF1 mice 3 days after transplantation, whereas CFSE-labelled BMMSCs were not detected (Fig. 1k). Immunostaining for human mitochondria (hMt) identified dDPSCs in the proximal colon 12 weeks after transplantation (Fig. 1l). The above results demonstrate that SDF1α-CXCR4 interactions play a crucial role in the migration of dDPSCs to regions of hypoganglionosis in JF1 mouse intestine.

### dDPSCs regenerate neurons and ICCs

**Figure 2:**
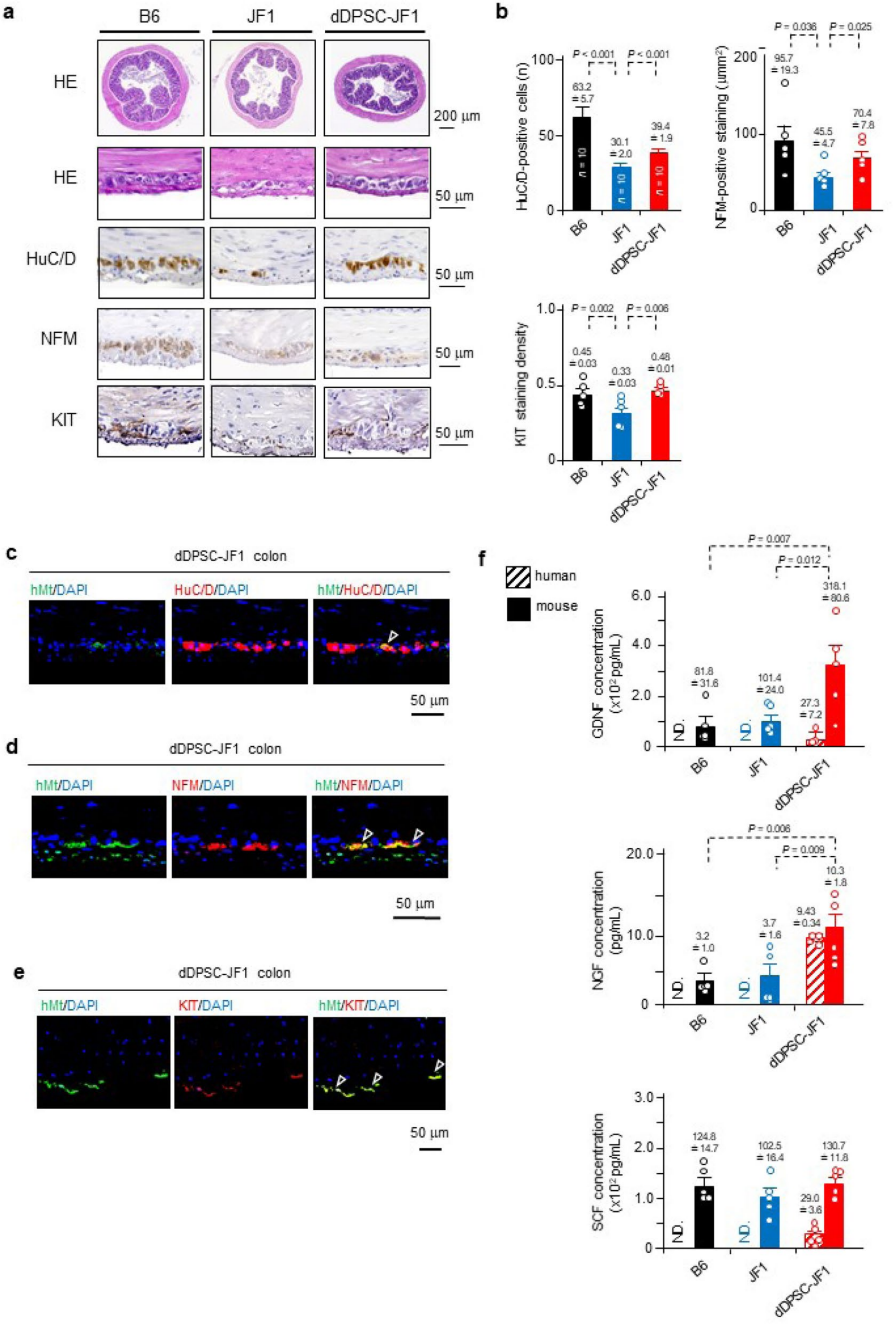
Differentiation of transplanted dDPSCs. (**a**) Proximal colon sections stained with haematoxylin–eosin (upper: × 40; lower: × 400) or immunostained for HuC/D (neuron-specific marker; × 400), NFM (peripheral ganglion marker; × 200) or KIT (pacemaker interstitial cell marker; × 200). (**b**) Number of HuC/D-positive cells per tissue cross-section (mean ± SEM, *n* = 10), NFM-positive area in the myenteric plexus (mean ± SEM, *n* = 5), and KIT-positive staining density in the proximal colon (mean ± SEM, *n* = 5). (**c–e**) Immunostaining for hMt, HuC/D, NFM and KIT (nuclei counterstained with DAPI). Arrowheads: dDPSC-derived cells. (**f**) Human and mouse NGF, GDNF and SCF concentrations (mean ± SEM, *n* = 5). ND, not detected. (**a,c–e**) Representative of ≥ 2 independent experiments.

Morphological investigations (Fig. 2a,b) revealed that JF1 mouse proximal colon exhibited poorer development of the intestinal musculature and myenteric region (haematoxylin–eosin staining), fewer myenteric ganglion cells (stained with the neuron-specific marker, HuC/HuD), and weaker immunostaining for neurofilament M (NFM, a peripheral ganglion marker) and KIT (a pacemaker interstitial cell marker). These observations suggest that congenital entero-neuropathy is accompanied by poor development of the pacemaker system^22,23^. Notably, dDPSC-JF1 mouse colon exhibited increased numbers of enteric neurons and pacemaker interstitial cells (Fig. 2a,b). Immunohistochemistry experiments in dDPSC-JF1 mice revealed that hMt was co-expressed in some HuC/HuD-positive and NFM-positive neurons in the myenteric plexus region (Fig. 2c,d) and in numerous KIT-positive cells (Fig. 2e), indicating that myenteric neurons and pacemaker cells had developed from dDPSCs.

Since inadequate interactions between trophic factors may contribute to hypoganglionosis^1–4^, we assessed the effects of dDPSCs on trophic factors. The concentrations of mouse nerve growth factor (NGF), glial cell-derived neurotrophic factor (GDNF) and stem cell factor (SCF, a KIT ligand) were similar between B6 and JF1 mice (Fig. 2f). dDPSC transplantation increased the protein levels of mouse NGF and GDNF and human GDNF, NGF and SCF (Fig. 2f). These findings are consistent with dDPSC-mediated paracrine actions.

### dDPSCs improve proximal colon function

We measured the mechanical and electrical activities of the second part of the proximal colon (Extended Data Fig. 1b) to assess the functional effects of dDPSC transplantation. Contractile responses to acetylcholine (Ach), endothelin-1 (ET-1) and electrical field stimulation (EFS) were smaller in JF1 mice than in B6 mice but comparable between dDPSC-JF1 mice and B6 mice (Extended Data Fig. 5a–d). The relaxation response to a nitric oxide (NO) donor was smaller in JF1 and dDPSC-JF1 mice than in B6 mice (Extended Data Fig. 5e). Contractile responses to a high concentration (60 mM) of extracellular K^+^ were similar among the three groups (Extended Data Fig. 5f), suggesting that the contractile ability of colonic smooth muscle was preserved in JF1 mice. The above findings agree well with the data in Fig. 2 (recovery of myenteric neurons after dDPSC transplantation).

**Figure 3:**
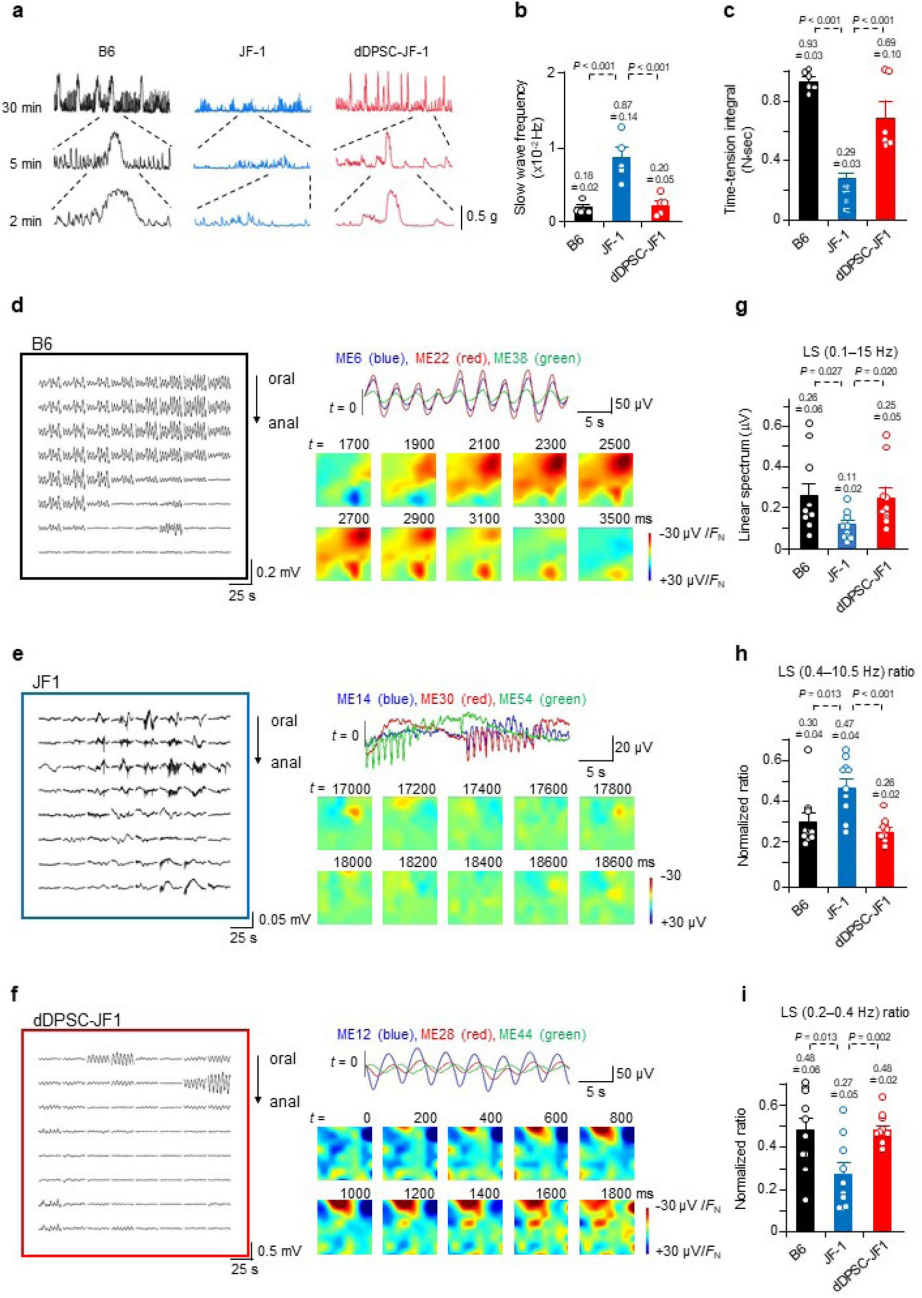
Effects of dDPSCs on colonic function. (**a**) Spontaneous contractions recorded from proximal colon muscle strips. (**b**,**c**) Slow wave frequency (mean ± SEM, *n* = 5) and time-tension integral (area under the curve) for the spontaneous contractions (mean ± SEM; B6, *n* = 6; JF1, *n* = 14; dDPSC-JF1, *n* = 7). (**d–f**) Electrical potentials recorded from the proximal colon with an 8 × 8 MEA and the corresponding reconstructed potential maps. (**g–i**) Characteristics of the electrical potentials recorded with the 8 × 8 MEA: potential magnitude, proportion of high-frequency (0.4–10.5 Hz) potentials, and proportion of low-frequency (0.2–0.4 Hz) potentials (mean ± SEM, *n* = 10). (**a,d–f**) Representative of ≥ 2 independent experiments.

We also evaluated the characteristics of spontaneous colonic contraction. The second part of the proximal colon exhibited irregular, high-frequency, small-amplitude, transient contractions in JF1 mice, which contrasted with the prolonged phasic contractions occurring at intervals of several minutes in B6 mice (Fig. 3a–c). dDPSC transplantation into JF1 mice restored the magnitude and frequency of spontaneous contractions to levels comparable to those in B6 mice (Fig. 3a–c). dDPSC transplantation also restored spontaneous contraction magnitude in the first part of the proximal colon (Extended Data Fig. 5g).

Spatiotemporally-coordinated electrical activity underlies the complex movements of the gastrointestinal tract^24^. We used the dialysis membrane-reinforced microelectrode array (MEA) technique to monitor field potentials in muscle sheets isolated from the second part of the proximal colon (Extended Data Fig. 6a–c). Samples from B6 mice displayed basal electrical oscillations with a period of 3.78 ± 0.22 s (*n* = 10 recording regions, *N* = 6 animals) in most of the recording area, and occasionally a large potential occurred (Fig. 3d and Extended Data Fig. 6d). Basal rhythmic oscillations rarely occurred in JF1 mouse colon (observed in 1/10 recording regions, *N* = 3), whereas electrical complexes of rapid and slow potentials occurred frequently (Fig. 3e and Extended Data Fig. 6e). The rapid potentials propagated in limited micro-regions (suggesting poor spatial synchronicity) with a frequency (1–2 Hz) comparable to that of myoelectric complexes^25–27^. dDPSC transplantation into JF1 mice restored the basal rhythmic oscillations, which occurred with an interval (4.12 ± 0.20 s, *n* = 10, *N* = 3) comparable to that in B6 mice (Fig. 3f and Extended Data Fig. 6f). However, the basal oscillation phase varied within the small recording area, and rapid potentials occurred locally in some samples, suggesting incomplete recovery of spatially-coordinated motor function. Spectral analysis (0.1–15 Hz) revealed that the electrical potential magnitude in JF1 mice was smaller than that in B6 mice but restored by dDPSC transplantation (Fig. 3g). The low-frequency component was smaller in JF1 mice than in B6 mice but comparable between dDPSC-JF1 and B6 mice (Fig. 3h,i), indicating that colons from dDPSC-JF1 and B6 mice exhibited similar regular basal rhythmicity. The effects of dDPSC transplantation on electrical characteristics are consistent with the restoration of myenteric neurons and pacemaker interstitial cells (Fig. 2).

### dDPSCs improve nutrition and survival

**Figure 4:**
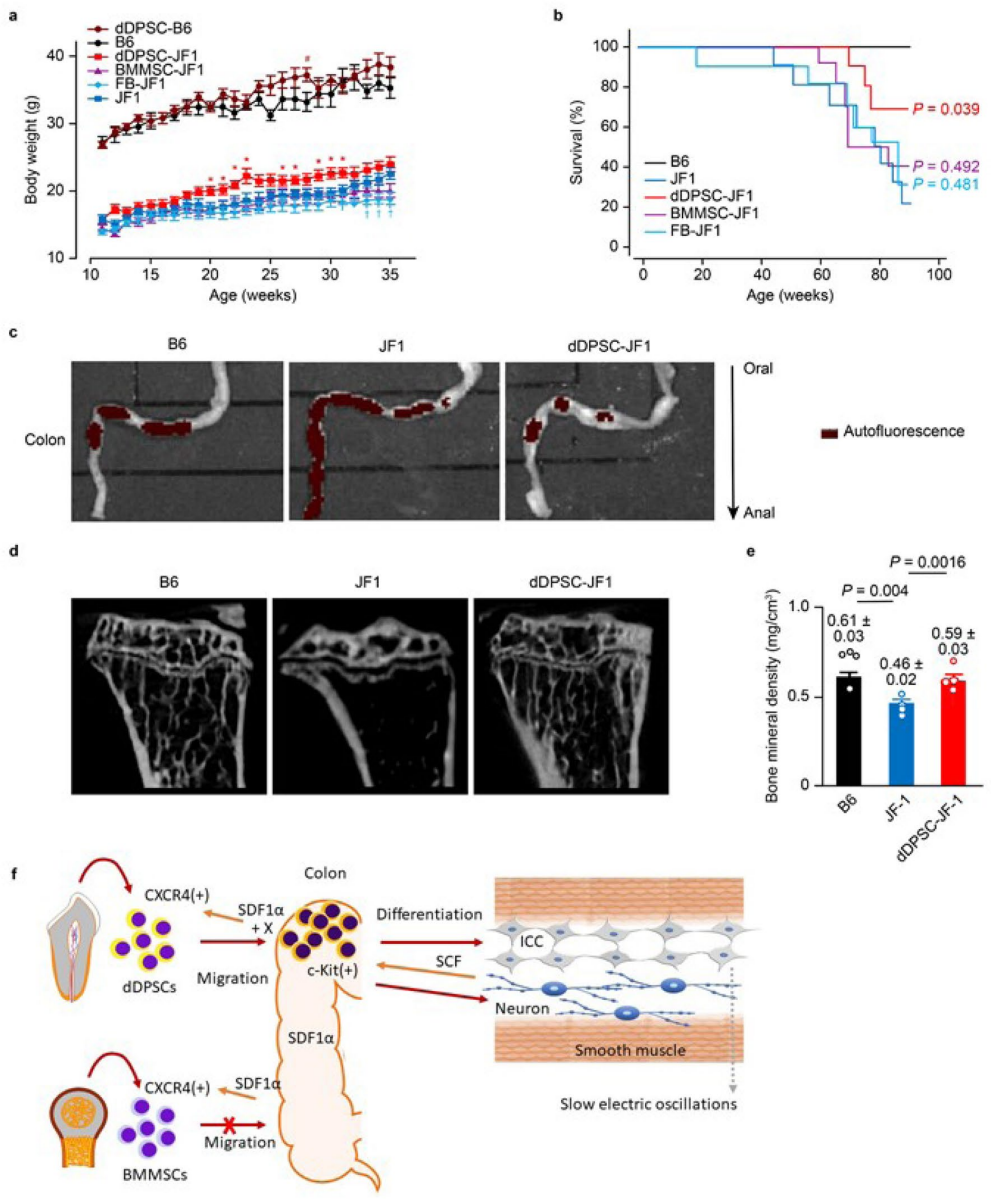
Therapeutic effects of dDPSCs. (**a**) Mouse bodyweight changes after the transplantation of stem cells into JF1 (mean ± SEM, *n* = 10) and B6 (mean ± SEM, *n* = 5) mice. (**b**) Kaplan–Meier survival curves (*n* = 10). Comparisons (vs. JF1 mice) were made using the log-rank test. (**c**) Autofluorescence of the colonic contents after a 24-h fast. Representative of ≥ 2 independent experiments. (**d**) Micro-CT of the tibia. (**e**) Tibial mineral density (mean ± SEM, *n* = 4). (**f**) Schematic diagram showing the possible mechanisms underlying dDPSC migration, differentiation and beneficial effects on colonic motility in JF1 mice.

JF1 mice exhibited slower bodyweight gain (from P11W to P35W) than B6 mice, and intravenous transplantation of dDPSCs (but not BMMSCs or FBs) caused a small but significant enhancement of bodyweight gain (during P20W to P31W: Fig. 4a and the photo in Extended Data Fig. 1a). Intraperitoneal injection of dDPSCs did not improve bodyweight gain in JF1 mice (data not shown). Median survival time (up to P90W) of JF1 mice was shorter than that of B6 mice (77.8 weeks vs. 112.2 weeks, *n* = 10, *P* = 0.004) and prolonged by the transplantation of dDPSCs (90.2 weeks, *n* = 10, *P* = 0.039 vs. JF1 mice) but not BMMSCs (80.0 weeks, *n* = 10, *P* = 0.492 vs. JF1 mice) or FBs (73.8 weeks, *n* = 10, *P* = 0.481 vs. JF1 mice; Fig. 4b). dDPSC transplantation into JF1 mice improved colonic transportation (ex vivo imaging of colonic content after a 24-h fast; Fig. 4c), restored long bone (tibial) mineral density (micro-computed tomography; Fig. 4d,e), enhanced hepatic glucose storage and improved the small intestinal mucosa (Extended Data Figs. 1c and 7) in P23W. These results indicate that multipotent dDPSCs can partially correct entero-neuropathy in JF1 mice (Fig. 4f), probably due to both their multi-lineage differentiation and paracrine effects.

Finally, we investigated potential off-target effects of dDPSC transplantation. dDPSC-JF1 mice exhibited no morphological alterations or T-cell accumulation in the lungs, liver or kidneys (Extended Data Fig. 8a) and no changes in the serum levels of enzymes related to the function of these organs (Extended Data Fig. 8b) or cytokines related to systemic inflammation (Extended Data Fig. 8c).

## Discussion

Animal models of spinal cord transection and autoimmune encephalitis have been treated with dDPSCs^28,29^, because these stem cells are known to differentiate into neural crest-derived cells, such as functional neurons and glial cells under appropriate conditions^30–32^. The treatment of congenital entero-neuropathies is more challenging because the complex cytoarchitecture of the gastrointestinal tract develops from three germ layers. Our study shows that dDPSCs have potential for development into a therapy for entero-neuropathies due to their multipotent characteristics. Intravenous administration of dDPSCs improved the survival and nutritional status (bodyweight and glucose uptake) of JF1 mice. We speculate that the latter effect was partly due to repair of the mucosa (endoderm) by epithelial cells generated by dDPSCs. Furthermore, staining for hMt indicated that dDPSCs differentiated into pacemaker interstitial cells (mesoderm) and enteric neurons (ectoderm). The ability of dDPSCs to undergo multi-lineage differentiation is critical for their therapeutic potential because numerous cells and mechanisms underlie coordinated gut motility^10^ and because congenital entero-neuropathies likely involve not only enteric neuron loss but also other impairments, as seen in JF1 mice. In addition, our data suggest that paracrine mechanisms contribute to the therapeutic effects of dDPSC transplantation, as seen in the treatment of the central nervous system^28,29^. In this study, although the body weight was temporarily improved (P20W to P31W) and the survival ratio was significantly prolonged, it was unverified whether dDPSCs differentiated into functional neurons and pacemaker cells, and how long they exist in recipients. In future study, to optimize the cell therapy of dDPSCs, these issues need to be addressed in relation with their paracrine effects and booster injections.

According to our observation, dDPSCs may have advantages over BMMSCs, FBs and other stem cell types in the treatment of refractory bowel disorders. Undifferentiated MSCs such as dDPSCs express high levels of inflammation-activated adhesion proteins that enables them to avoid mechanical trapping in healthy capillaries and settle in regions affected by inflammation^33^. The lack of intestinal accumulation of dDPSCs in B6 and anti-SDF1α antibody-treated JF1 mice demonstrates the crucial role of SDF1α-CXCR4 interactions in the migration of dDPSCs to affected bowel regions. However, CXCR4-expressing BMMSCs did not colonize the myenteric region or improve the nutritional status of JF1 mice, indicating that mechanisms other than SDF1α-CXCR4 interactions contribute to the migration and colonization processes. For example, dDPSCs express KIT whereas BMMSCs do not, and it has been reported that SCF (a KIT ligand released from enteric neurons and smooth muscle cells) induces the differentiation of MSC-derived progenitor cells into ICCs^34^. It is likely that transplanted dDPSCs differentiate into progenitors of enteric neurons and pacemaker cells, which act synergistically to correct bowel dysmotility through interactions between SCF and KIT and the release of neurotransmitters and hormones that promote the development of ICCs^34^.

Various cell types have been evaluated for possible use as a cell-based therapy for HSCR and its allied disorders. Enteric NCC-derived progenitors from mice successfully develop into functional neurons when grafted into the colon of wild-type recipient mice with the same background^35^. Moreover, pluripotent stem cells such as embryonic stem cells (ESCs) and induced pluripotent stem cells (iPSCs) can regenerate gut-like organoids that exhibit neural responses and pacemaker potentials^36,37^. Recently, an ESC-derived NCC lineage was shown to engraft successfully and reduce disease-related mortality in an animal model of HSCR (mice with an *Ednrb* mutation)^38^. As with dDPSCs, the therapeutic effect of NCCs derived from pluripotent stem cells may be enhanced by the simultaneous development of pacemaker cell lineages. However, the clinical application of ESC- and iPSC-derived lineages is currently controversial due to possible carcinogenic and immunogenic effects^39,40^, which highlights the low immunogenicity of dDPSCs as an important advantage (Extended Data Fig. 3). We observed hMt-containing neurons and pacemaker cells in the colon of JF1 mice 12 weeks after transplantation as well as an improvement in both mechanical and electrical function (Figs. 2 and 3). Moreover, the high expression of phosphatase and tensin homolog (PTEN) is thought to reduce the carcinogenicity of dental pulp stem cells^41^. Although autologous transplantation would be preferable for the clinical application of dDPSCs, the low immunogenicity of these cells would likely allow allogeneic transplantation of dDPSCs obtained from deciduous or extracted teeth.

The colon exhibits characteristic patterns of electrical potentials. Slowly oscillating potentials (occurring at intervals of 3–4 s) are prominent in the proximal colon of wild-type mice. KIT-positive pacemaker cells are the likely origin of slow potentials^22,23,42^. Spontaneous myoelectric complexes comprising rapid and slow components occur in the mid-to-distal colon, and this electrical activity is considered neurogenic in origin and the basis of propulsion^27^. Activity resembling myoelectric complexes is observed in JF1 mouse proximal colon despite the poor spatial synchronicity, suggesting that loss of spontaneous rhythmicity generated by network-forming pacemaker cells is compensated for by the relatively preserved enteric neurons. We propose that both the ENS and network-forming pacemaker system are impaired in entero-neuropathies. This hypothesis would account for some of the observed discrepancies in colonic motility, such as bi-directional propagation of colonic migrating motor complexes^26^.

Heterozygous and homozygous *sl* mutations of *Ednrb* cause similar reductions in the myenteric ganglia of mice, but the homozygous *sl* mutation is lethal due to a complete absence of motility^43^, which would suggest a role for factors other than ENS impairment. Mutations of *Ret* are known to be associated with defects in enteric neurons, and homozygous mutations of *Ret* also yield HSCR-like mice, whereas the heterozygous mutation is asymptomatic. Recent studies of the *Ret*-null mouse embryo suggest that *Ret* has much broader effects than enteric neurogenesis^44^, as we suggest for the *Ednrb* mutation. We speculate that symptoms of bowel dysmotility in animal models of congenital entero-neuropathies arise from the additional impairment of the pacemaker system, because coordinated gastrointestinal movement is maintained by a multitude of cooperating systems. This may also be true in humans, particularly given the discrepancy between bowel motility symptoms and morphological defects in the ENS.

In conclusion, the pacemaker and intrinsic nervous systems of the proximal colon are impaired in JF1 mice with *Ednrb* mutation. Therefore, appropriate cell-based therapy seems to require stem cells with multi-lineage potential as well as their paracrine effects in this model of gut motility disorder. Notably, transplanted dDPSCs migrate to affected regions of the colon and improve the electrical and mechanical activity, likely because they differentiate into pacemaker interstitial cells and enteric neurons. Further investigations are required to optimise the transplantation technique, for example by improving cell migration to affected intestinal regions and promoting cell differentiation to repair defective tissues. Nevertheless, we consider dDPSC transplantation as a promising cell-based therapy for congenital entero-neuropathies, especially for patients with pacemaker dysfunction in apparently ‘healthy’ intestinal segments.

## Methods

### Ethics statement

All methods were performed in accordance with relevant guidelines and regulations for humans and animals. All authors agreed that each is responsible for the accuracy and fairness of the related research.

Healthy human deciduous teeth were collected as discarded biological/clinical samples from three healthy donors (5–7 years old) who attended the Department of Pediatric Dentistry of Kyushu University Hospital. The three samples were randomized to use in each experiment. The parents/guardians of the donors provided written informed consent that stem cells harvested from the teeth could be used for research into regenerative medicine for congenital diseases. Human peripheral blood mononuclear cells (PBMNCs) were collected from peripheral venous blood obtained from donors (25–28 years old) unrelated to those who provided deciduous teeth. Informed consent was obtained from all subjects for use of Human PBMNCs. Procedures for handling human samples were approved by Kyushu University Institutional Review Board for Human Genome/Gene Research (Protocol Number: 393-00).

All animal experiments were approved by the Institutional Animal Care and Use Committee of Kyushu University (Protocol Number: A21-044-1; A25-086–0) and Nagoya University (Permissions #28316, #29121, #30312, #31199 and #20430). Stem cell transplantation caused no substantial biological alterations or welfare issues in the animals (Fig. 4 and Extended Data Fig. 8). The study was carried out in compliance with ARRIVE guidelines.

### Animals

Male JF1 mice (National Institute of Genetics, Mishima, Japan) and male and female wild-type C57BL/6J (B6) mice (Kyudo, Tosu, Japan; Japan SLC, Hamamatsu, Japan) were housed under controlled environmental conditions (12-h light/12-h dark cycle) and given free access to sterile drinking water and a standard MF chow diet (Oriental Yeast, Tokyo, Japan).

### Stem cell isolation and culture

Isolation and culture of stem cells from dental pulp tissue were performed using enzyme digestion and colony-forming unit fibroblast (CFU-F) methods^14,15,45,46^. Remnant dental pulp tissues from extracted human deciduous teeth were digested with 0.3% collagenase type I (Worthington Biochemicals, Lakewood, NJ, USA) and 0.4% dispase II (Sanko Junyaku Co., Ltd., Tokyo, Japan) for 60 min at 37 °C. The resultant cells were passed through a 70-µm cell strainer and seeded on T-75 culture flasks. Three hours after cell seeding, the culture flasks were washed with sterilized Ca^2+^-free and Mg^2+^-free phosphate-buffered saline (PBS). The remaining adherent cells were incubated in Minimum Essential Medium Eagle-Alpha Modification (αMEM; Thermo Fisher Scientific, Waltham, MA, USA) containing 15% foetal bovine serum (FBS; Equitech-Bio, Kerrville, TX, USA), 100 µM l-ascorbic acid 2-phosphate (Wako Pure Chemicals, Osaka, Japan), 2 mM l-glutamine (Nacalai Tesque, Kyoto, Japan) and premixed 100 U/mL penicillin/100 µg/mL streptomycin (Nacalai Tesque) for 16 days. The resulting colonies were passaged to expand the number of dDPSCs. Cells at P3 were used for the experiments.

BMMSCs isolated from human whole bone marrow aspirates obtained from healthy adult volunteers (*n* = 3; AllCells, Alameda, CA, USA) and human skin fibroblasts (FBs; PromoCell, Heidelberg, Germany) were prepared as described previously^15^.

### Stem cell characterization

dDPSCs were characterized according to standard protocols^47^. The adherent colony-forming capacity was evaluated using a CFU-F assay. Isolated cells (1 × 10^4^) were seeded on T-75 culture flasks and cultured in growth medium for 16 days. The flasks were treated with 4% paraformaldehyde and 0.1% toluidine blue in PBS (pH 7.4) for 18 h. The number of fibroblast colonies containing > 50 cells was counted under a microscope.

The multipotency of dDPSCs (P3) was verified according to previous studies^12,15^. Briefly, specific culture conditions were used to induce dDPSCs to differentiate into osteoblasts, chondrocytes or adipocytes that were stained with alizarin red-S, toluidine blue or oil red-O, respectively. Expressions of genes specific to osteoblasts (runt-related transcription factor-2 [RUNX2] and osteocalcin [BGLAP]), chondrocytes (SRY-box-9 [SOX9] and aggrecan [ACAN]) and adipocytes (peroxisome proliferator-activated receptor gamma-2 [PPARG] and lipoprotein lipase [LPL]) were analysed by quantitative reverse transcription polymerase chain reaction (RT-qPCR). Expressions of genes specific for neural crest cells, including nerve growth factor receptor (NGFR), paired box-3 (PAX3), zinc finger protein SNAI-2 (SLUG), zinc finger protein SNAI-1 (SNAIL), SOX9 and endothelin receptor type B (EDNRB), in dDPSCs (P3) were examined by RT-qPCR. Expressions of CXCR4 and KIT in dDPSCs (P3) were assessed using immunofluorescence experiments (see Supplementary Table 1). We have previously confirmed that cultured dDPSCs express stem cell markers (positive for CD146, CD73, CD105, and CD90, but negative for hematopoietic markers of CD34, CD45, CD14, and CD11b) and low levels of class II HLA and T cell costimulatory molecules^46^.

For mixed lymphocyte assays, human PBMNCs (1 × 10^6^) were co-cultured with dDPSCs (1 × 10^5^) and gamma-irradiated human PBMNCs (1 × 10^5^; 30 Gy irradiation with an MBR-1520R-3 irradiator; Hitachi, Tokyo, Japan) or stimulated with phytohemagglutinin (5 μg/mL; Merck, Darmstadt, Germany) in RPMI-1640 medium (Sigma-Aldrich) containing 10% heat-inactivated FBS (Equitech-Bio), 2 mM l-glutamine, 1 mM sodium pyruvate and premixed 100 U/mL penicillin/100 µg/mL streptomycin (Nacalai Tesque). Five days after co-culture or stimulation, cell viability was assayed using Cell Counting Kit-8 (Dojindo, Kumamoto, Japan) and a Multiscan GO plate reader (Thermo Fisher Scientific).

### Stem cell transplantation

dDPSCs, BMMSCs or FBs (10^4^ cells/g bodyweight in 100 µL PBS) were intravenously injected into JF1 mice at P11W, by reference to previous transplantation procedures in wild-type mice^15^. Age-matched B6 and JF1 mice intravenously injected with PBS (100 µL) were used as controls. Some JF1 mice were injected intraperitoneally with anti-SDF1α antibody (100 µg in 100 µL PBS; R&D Systems, Minneapolis, MN, USA) or PBS (100 µL) 24 h before transplantation.

Mice were divided into five experimental groups: B6 (*n* = 10), (PBS-infused) JF1 (*n* = 10), dDPSC-transplanted JF1 (*n* = 10), BMMSC-transplanted JF1 (*n* = 10) and FB-transplanted JF1 (*n* = 10). Mouse bodyweight, water intake, food intake and stool number were measured weekly from P11W to P35W. Some mice were selected randomly for harvesting of organ tissues and peripheral blood at P23W (12 weeks after transplantation), and the intestine was dissected into lengths of ~ 15 mm. Mice were sacrificed by cervical dislocation after deeply anesthetized with inhaled isoflurane (Kyushu University), or were euthanized by cervical dislocation after respiratory arrest with inhaled diethyl ether/carbon dioxide (Nagoya University).

### Monitoring of transplanted cells

To reduce the background autofluorescence of standard chow, mice were fed with alfalfa-free Ivid#2 feed (Oriental Yeast) for 10 days before in vivo and ex vivo imaging. dDPSCs were incubated with DiR (Perkin Elmer, Waltham, MA, USA) for 30 min at 37 °C and washed twice with PBS. Fluorescent dDPSCs (10^5^ cells/g bodyweight in 100 µL PBS) were intravenously transplanted into recipient mice (P23W). PBS-infused mice were used as controls. Whole-body images and ex vivo images of organs were acquired 1, 7 and 20 days after transplantation using an IVIS Lumina III system (Perkin Elmer). In the experiment involving the injection of anti-SDF1α antibody into a JF1 mouse, ex vivo imaging was performed 18 days after dDPSC transplantation due to early death of the mouse. Colour images showing DiR fluorescence above a threshold level were superimposed on greyscale images of the mouse body or resected organ.

### Peristalsis

Mice were fed with standard chow, which exhibits autofluorescence. The entire gastrointestinal tract was resected (P23W) after a 24-h fast. The autofluorescence of the intestinal contents was measured from ex vivo images acquired with an IVIS Lumina III system (Perkin Elmer). A reduction in autofluorescence level was interpreted as an increase in peristaltic movements.

### Nutritional status

Improvements in nutritional status (hepatic glucose uptake and body weight) after dDPSC transplantation were used as surrogate markers of mucosal repair in the small intestine. Tibial bones were harvested from each experimental group at P23W, and the distal tibial epiphyses were imaged using a Skyscan 1076 micro-CT system (Skyscan, Kontich, Belgium). Bone mineral density (BMD) and bone parameters were estimated using CT-Analyzer software (Skyscan). BMD values were calibrated using hydroxyapatite phantoms with BMD values of 0.25 and 0.75 g/cm^3^ (Skyscan).

In vivo hepatic glucose uptake was measured in mice (P23W) given free access to water containing 1% 2-deoxy-D-glucose (2DG; Merck)^48^. Liver tissue samples were collected after 7 days. Hepatic 2DG content (an index of glucose storage) was assayed using a commercial kit (Cosmo Bio, Otaru, Japan) and plate reader (Multiscan GO; Thermo Fisher Scientific).

### Histology and immunohistochemistry

Samples were fixed with 4% paraformaldehyde in PBS (pH 7.2) at 4 °C overnight, dehydrated, cleaned and embedded in paraffin. For histology, sections were stained with haematoxylin–eosin. For immunohistochemistry, sections were incubated first with 3% H_2_O_2_ in ethanol for 30 min to inhibit endogenous peroxidase and then with 10% bovine serum albumin in PBS for 60 min. Sections were treated with 0.01 M citrate buffer (pH 6.0 or 9.0) in a microwave processor (MI-77, Azumaya, Tokyo, Japan) for antigen retrieval. Sections were incubated with primary antibodies (Supplementary Table 2) at 4 °C overnight. Visualisation was achieved using an EnVision + System (Agilent, Santa Clara, CA, USA). Sections were lightly counterstained with haematoxylin. For double-immunofluorescence staining, sections were incubated with primary antibodies (Supplementary Table S2) and then treated with the Opal 3-plex kit (Perkin Elmer). Sections were mounted onto slides using Vectashield Mounting Medium with DAPI (Vector Laboratories, Burlingame, CA, USA). Negative controls utilized isotype-matched antibodies instead of primary antibodies. Images were obtained using a BZ-X700 fluorescence microscope (Keyence, Tokyo, Japan). The number and area of ganglion cells in myenteric ganglia were estimated using Hybrid Cell Count software (Keyence)^49–51^.

### Quantitative assays

Blood samples were incubated overnight at 4 °C and centrifuged at 1,500 rpm for 10 min at 4 °C to obtain serum. Tissue samples (including liver, lung and kidney) were homogenized in T-PER Tissue Protein Extraction Reagent (Thermo Fisher Scientific) containing a proteinase inhibitor cocktail (Nacalai Tesque) using a Vibra-Cell ultrasonic processor (Sonic & Materials, Newtown, CT, USA). Total protein was measured using a protein assay (Bio-Rad Laboratories, Hercules, CA, USA) and normalized to the tissue sample weight.

Commercial kits were used to measure serum levels of creatinine (Creatinine Colorimetric Assay kit, Cayman Chemical Company, Ann Arbor, MI, USA), alanine aminotransferase and aspartate aminotransferase (Transaminase CII-Test kit, Wako Pure Chemicals). Commercial enzyme-linked immunosorbent assay (ELISA) kits were used to determine albumin, GDNF, IL-2, MIP-2, NGF, SCF and SDF1α protein levels in mouse tissues or serum (see Supplementary Table 3). The colourimetric assay and ELISA results were analysed using a Multiscan GO plate reader (Thermo Fisher Scientific).

Serum levels of IL-1α, IL-1β, IL-2, IL-5, IL-6, IL-12, IL-18, granulocyte-colony stimulating factor, tumour necrosis factor-α, interferon-γ and macrophage inflammatory protein-1β (MIP1β) were quantified using a Bio-Plex MAGPIX system and a Bio-Plex Pro multiplex cytokine assay system (Bio-Rad Laboratories).

### RT-qPCR

Samples were treated with TRIzol (Thermo Fisher Scientific). RNA extracts were digested with DNase I (Promega, Fitchburg, WI, USA) and purified using the RNeasy Mini Kit (Qiagen, Venlo, Netherlands). Total RNA samples were treated with a Revertra Ace qPCR kit (Toyobo, Osaka, Japan) to obtain cDNA. cDNA samples were mixed with EagleTaq Universal Master Mix (Roche, Basel, Switzerland) and target-specific TaqMan probes (Thermo Fisher Scientific; Supplementary Tables 4 and 5). RT-qPCR was performed using a Light Cycler 96 system (Roche) under the following conditions: 50 °C for 120 s; 95 °C for 600 s; 45 cycles of 95 °C for 15 s and 60 °C for 60 s. Glyceraldehyde 3-phosphate dehydrogenase mRNA (mouse samples) and 18S ribosomal RNA (human samples) were used for data normalization.

### Mouse intestinal microbiota

Stools were collected daily during P24W–P26W. Faecal bacterial DNA content was analysed by the Primary Cell Division of Cosmo Bio (Sapporo, Japan; protocol #151228A1 and #160125A1). Briefly, microbial DNA was extracted from the faecal sample using the Isospin Fecal DNA kit (Nippon Gene, Tokyo, Japan). The V3V4 region of the 16S rRNA gene was amplified (Extended Data Fig. 2a,b: Repertoire Genesis, Ibaraki, Japan) by PCR (forward: CCTACGGGNGGCWGCAG; reverse: GACTACHVGGGTATCTAATCC) and sequenced using a MiSeq Deep sequencer and MiSeq Reagent Kit v3 (Illumina, San Diego, CA, USA). The sequence data were pre-processed and analysed using Flora Genesis software (Extended Data Fig. 2c: Repertoire Genesis, Ibaraki, Japan). The R1 and R2 read pairs were joined, and chimera sequences were removed. Open-reference operational taxonomic unit (OTU) picking was performed using the 97% ID prefiltered Greengenes database and Uclust. Representative sequences of each OTU were chosen, and taxonomy assignment was performed using the Ribosomal Database Project (RDP) classifier and a threshold score ≥ 0.5. OTUs were grouped if their annotation was the same regardless of RDP score. Bacterial floral communities were characterised using the Gini-Simpson index (1/D)^52,53^.

### Colonic mechanical activity

Mechanical responses to pharmacological agents and EFS were measured in 15-mm-long muscle strips isolated from the proximal colon. Macroscopically, the mouse proximal colon has two bulging regions; here we refer to the oral one as the first part and the anal one as the second part. The second part of the proximal colon was used in all experiments except those shown in Extended Data Fig. 5g. The modified Krebs solution consisted of 121.6 mM NaCl, 4.7 mM KCl, 2.5 mM CaCl_2_, 1.2 mM MgCl_2_, 1.2 mM Na_2_KH_2_PO_4_, 15.4 mM NaHCO_3_ and 11.5 mM D-glucose gassed with 5% CO_2_ and 95% O_2_ (pH 7.4 at 37 °C). The strips were mounted vertically in an organ bath maintained at 37 °C. Changes in isometric tension generated by the circular muscle were recorded using a force transducer (TB-612 T; Nihon Kohden, Tokyo, Japan) and amplifier (AS1202; NEC, Tokyo, Japan) and analysed using LabChart and Scope software (ADInstruments, Bella Vista, NSW, Australia). Enteric nerve stimulation was achieved using EFS (50 V, 0.5-ms step pulses repeated at 5 Hz for 5 s) applied by an electrical stimulator (SEN-3301; Nihon Kohden). The following pharmacological agents were used: 1 µM Ach (Wako Pure Chemicals), 10 nM ET-1 (Wako Pure Chemicals), 10 µM NO (Wako Pure Chemicals), 60 mM KCl, 1 µM atropine sulphate (Wako Pure Chemicals; to block the response to Ach) and 1 µM tetrodotoxin (Wako Pure Chemicals; to block the response to EFS). Tension development in response to EFS and pharmacological agents was normalized to the tissue weight and response to 60 mM KCl.

### Colonic electrical activity

The spatiotemporal characteristics of proximal colon electrical activity were assessed using an 8 × 8 MEA recording system (Alpha MED Scientific, Ibaraki, Japan)^54,55^. Colonic smooth muscle samples were prepared by gently removing the mucosa. Each sample was mounted on an 8 × 8 MEA (interpolar distance, 150 μm) with the longitudinal muscle layer facing downward using a piece of dialysis membrane fixed with a slice anchor (SDH series, Harvard Apparatus Japan, Tokyo, Japan; Extended Data Fig. 6). The 2-mL recording chamber was perfused (1–2 mL/min at 34 °C) with modified Krebs solution containing HEPES/Tris pH-buffer instead of bicarbonate to prevent the development of microbubbles on the MEA. The sample was perfused with solution for ~ 30 min before recording was started, and perfusion was discontinued during recording to minimize electrical noise. A set of 8 × 8 field potentials were simultaneously recorded using a computer-controlled, multi-channel AC amplifier (low-pass filtering at 10 kHz) and 14-bit A/D converters (sampling rate of 20 kHz). The dynamic range of A/D conversion was usually ± 1 mV. High-pass (0.1 Hz) filtering was applied to stabilize the baseline drift of the microelectrode potential.

A low-impedance MEA was employed to measure a wide range of electrical activity, including pacemaker potentials occurring at ~ 4-s intervals and myoelectric complex-like potentials with rapid and slow components^54^. Each recording electrode was a square (~ 50 μm × 50 μm) made from platinum black nanoparticles, which increased the surface area by ~ 200-fold to 0.5 mm^2^. The capacitance (CME) and resistance (RME) of each microelectrode was 0.052 μF and 15 kΩ, respectively^54,56^, hence the impedance of the recording electrode at 0.1 Hz was estimated to be small enough [~ 31 MΩ = √ {1/(2π × 0.1 Hz × 0.052 μF)^2^ + (15 kΩ)^2^}] to follow oscillating potentials when compared to the input impedance of the multi-channel amplifier (100 MΩ at 0.1 Hz). The efficacy of electrical signal transmission (Tr) was estimated to be ~ 95% at 0.1 Hz [100 MΩ / √ {(100 MΩ)^2^ + (31 MΩ)^2^}]. Moreover, the low resistance (15 kΩ) of the electrodes was advantageous for reducing thermal noise (NT), which was calculated to be ~ 1.6 μV at the low-pass frequency (10 kHz) of the AC amplifier.

To generate pseudocolour images, the field potential data were thinned 100–1000-fold in the time domain and bandpass filtered at 0.25–30 Hz (Kaiseki Excel add-in software, Kyowa, Tokyo, Japan). At certain recording times, the 8 × 8 field potentials were interpolated by a spline function with 50 points between each potential using MATLAB (MathWorks, Natick, MA, USA)^53^. To reconstitute smooth potential images, the amplitude of the field potential in each ME was compensated by the linear spectrum at 0.1–15 Hz^54^. The upper and lower images corresponded to the oral and anal ends of the MEA recording region, respectively. Field potential videos were produced at a frame rate of 200 Hz.

### Statistical analysis

Data are expressed as the mean ± standard error of the mean (SEM) of at least three determinations. Inter-group comparisons were made using two-tailed Student’s t-tests for distinct samples (two groups), Mann–Whitney U-test or one-way repeated-measures analysis of variance (ANOVA) followed by the Tukey post-hoc test (three or more groups). Kaplan–Meier analyses and log-rank tests were used for survival analyses. *P* < 0.05 was considered significant. Analyses were performed using JMP 11 (SAS Institute, Cary, NC, USA).

## Supplementary Information

Supplementary Video 1.

Supplementary Video 2.

Supplementary Video 3.

Supplementary Information 1.

## Data Availability

The data that support the findings of this study are available from the corresponding author upon reasonable request. Source data for figures are provided within this paper.
