# Supplementary material for: Dental pulp stem cells as a therapy for congenital entero-neuropathy
Source: Sci Rep. 2022 Apr 28;12:6990. doi: 10.1038/s41598-022-10077-3 (PMC9051124; doi:10.1038/s41598-022-10077-3)
Supplement: Supplementary file 4 — Supplementary Information 1. [file 41598_2022_10077_MOESM4_ESM.doc]

**Dental pulp stem cells as a therapy for congenital entero-neuropathy**

**Supplementary Information**

**Supplementary Video1:** Fig3d_WT_16.5-26.5s_BPF_Norm_max30.avi.

Video showing field potential maps (t = 16.5 to 26.5 s) in a wild-type B6 mouse corresponding to Fig.3d. MEA field potentials were bandpass filtered and normalized by using linear spectrum.

**Supplementary Video2:** Fig3e_JF1_16-26s_BPF_Norm_max30.avi.

Video showing field potential maps (t = 16 to 26 s) in a JF1 mouse corresponding to Fig.3e. MEA field potentials were bandpass filtered and normalized by using linear spectrum.

**Supplementary Video3:** Fig3f_dDPSC-JF1_-2-8s_BPF_Norm_max30.avi.

Video showing field potential maps (t = -2 to 8 s) in a dDPSC-JF1 mouse corresponding to Fig.3f. MEA field potentials were bandpass filtered and normalized by using linear spectrum.

**Supplementary Table 1. List of fluorochrome-conjugated antibodies used to detect stem cells.**

| **Name (clone)** | **Type** | **Manufacturer** |
| --- | --- | --- |
| Anti-CXCR4 antibody (12G5) | R-PE-conjugated, mouse IgG2a, kappa | Biolegend (San Diego, CA, USA) |
| Anti-KIT antibody (104D2) | R-PE-conjugated, mouse IgG1, kappa | Biolegend (San Diego, CA, USA) |
| Mouse IgG1 kappa (MOPC-21) | R-PE-conjugated | Biolegend (San Diego, CA, USA) |
| Mouse IgG2a kappa (MOPC-173) | R-PE-conjugated | Biolegend (San Diego, CA, USA) |
| Mouse IgG2b kappa (MPC-11) | R-PE-conjugated | Biolegend (San Diego, CA, USA) |

CXCR4, C-X-C chemokine receptor type 4; IgG, immunoglobulin G; R-PE: R-phycoerythrin.

**Supplementary Table 2. Primary antibodies used for immunohistochemistry**.

| **Name (clone)** | **Type** | **Manufacturer** | **Catalogue no.** |
| --- | --- | --- | --- |
| Anti-CD3 | Rabbit IgG | Abcam (Cambridge, UK) | ab5690 |
| Anti-ETBR | Rabbit IgG | LifeSpan BioSciences (Seattle, WA, USA) | LS-A54 |
| Anti-F4/80 (BM8) | Mouse IgG2b, kappa | Thermo Fisher Scientific, (Waltham, MA, USA) | 14-4801 |
| Anti-HuC/HuD (16A11) | Mouse IgG2b, kappa | Thermo Fisher Scientific, (Waltham, MA, USA) | A-21271 |
| Anti-KIT | Rabbit IgG | Agilent (Santa Clara, CA, USA) | A4502 |
| Anti-mitochondria, human (113-1) | Mouse IgG1 | Abcam (Cambridge, UK) | 113-1 |
| Anti-NFM antibody (RMO-270) | Mouse IgG2b, kappa | Thermo Fisher Scientific, (Waltham, MA, USA) | 13-0700 |
| Anti-SFD1 antibody | Rabbit IgG | Abcam (Cambridge, UK) | ab25117 |
| Mouse IgG1 kappa (MOPC-21) | Mouse IgG1, kappa | Biolegend (San Diego, CA, USA) | 400111 |
| Mouse IgG2b kappa (MPC-11) | Mouse IgG2b, kappa | Biolegend (San Diego, CA, USA) | 400319 |
| Rabbit IgG | Rabbit IgG | Biolegend (San Diego, CA, USA) | 410404 |

ETBR, endothelin receptor type B; IgG, immunoglobulin G; NFM, neurofilament M; SFD1, stromal cell-derived factor-1.

**Supplementary Table 3. List of commercially available kits used for enzyme-linked immunosorbent assays**.

| **Target protein** | **Product** | **Manufacturer** | **Catalogue no.** |
| --- | --- | --- | --- |
| Albumin, mouse | Mouse Albumin ELISA Quantitation Kit | Bethyl Laboratories (Montgomery, TX, USA) | E90/99-134 |
| DAO, mouse | IDK DAO ELISA | Immundiagnostik AG (Bensheim, Germany) | K8500 |
| Endotoxin | Limulus Amebocyte Lysate Chromogenic Endpoint Assay | Hycult Biotech (Uden, Netherlands) | HIT302 |
| GDNF, human | GDNF Human ELISA Kit | Abcam (Cambridge, UK) | ab100525 |
| GDNF, mouse | GDNF Mouse ELISA Kit | Abcam (Cambridge, UK) | ab171178 |
| KIT, human | Quantikine ELISA Human CD117/c-kit Immunoassay | R&D Systems (Minneapolis, MN, USA) | DSCR00 |
| KIT, mouse | c-Kit (CD117) Mouse SimpleStep ELISA Kit | Abcam (Cambridge, UK) | ab203361 |
| NGF, human | Human nerve growth factor (NGF) ELISA Kit | Cusabio (Houston, TX, USA) | CSB-E04683h |
| NGF, mouse | Mouse nerve growth factor (NGF) ELISA Kit | Cusabio (Houston, TX, USA) | CSB-E04684m |
| SCF, human | Quantikine ELISA Human SCF Immunoassay | R&D Systems (Minneapolis, MN, USA) | DCK00 |
| SCF, mouse | Quantikine ELISA Mouse SCF Immunoassay | R&D Systems (Minneapolis, MN, USA) | MCK00 |
| SDF1α, mouse | Quantikine ELISA Mouse CXCL12/SDF-1α Immunoassay | R&D Systems (Minneapolis, MN, USA) | MCX120 |
| SP-D, mouse | Quantikine ELISA Mouse SP-D Immunoassay | R&D Systems (Minneapolis, MN, USA) | MSFPDO |

DAO, diamine oxidase; GDNF, glial cell-derived neurotrophic factor; NGF, nerve growth factor; SCF, stem cell factor; SDF1α, stromal cell-derived factor 1α; SP-D, surfactant protein D.

**Supplementary Table 4. List of TaqMan probes for mouse genes used for RT-qPCR.**

| **Symbol** | **Name** | **ID number** |
| --- | --- | --- |
| *Ednrb* | Endothelin receptor type B | Mm00432989_m1 |
| *Gapdh* | Glyceraldehyde 3-phosphate dehydrogenase | Mm99999915_g1 |
| *Kit* | Kit oncogene (CD117) | Mm00445212_m1 |

**Supplementary Table 5. List of TaqMan probes to human genes used for RT-qPCR.**

| **Symbol** | **Name** | **ID number** |
| --- | --- | --- |
| ACAN | Aggrecan | Hs00153936_m1 |
| BGLAP | Bone gamma-carboxyglutamate protein | Hs01587814_g1 |
| LPL | Lipoprotein lipase | Hs00173425_m1 |
| PPARG | Peroxisome proliferator-activated receptor-gamma | Hs0115513_m1 |
| RUNX2 | Runt-related transcription factor-2 | Hs00231692_m1 |
| SOX9 | SRY-box 9 | Hs01001343_g1 |
| 18S | Eukaryotic 18S ribosomal RNA | Hs99999901_s1 |

**Supplementary Image 1. The whole gel used in Extended Data Fig. 2a.**

**
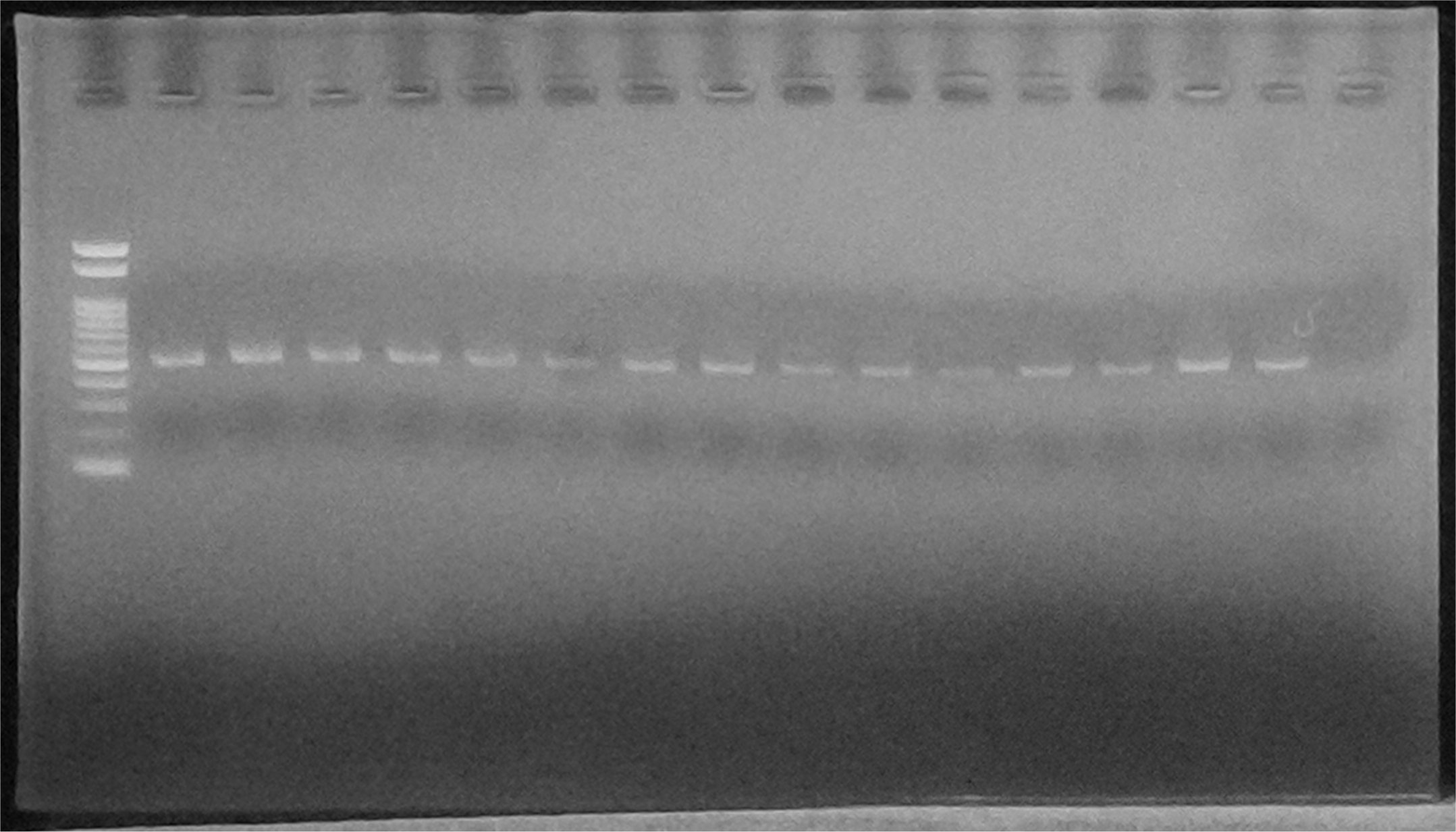
**


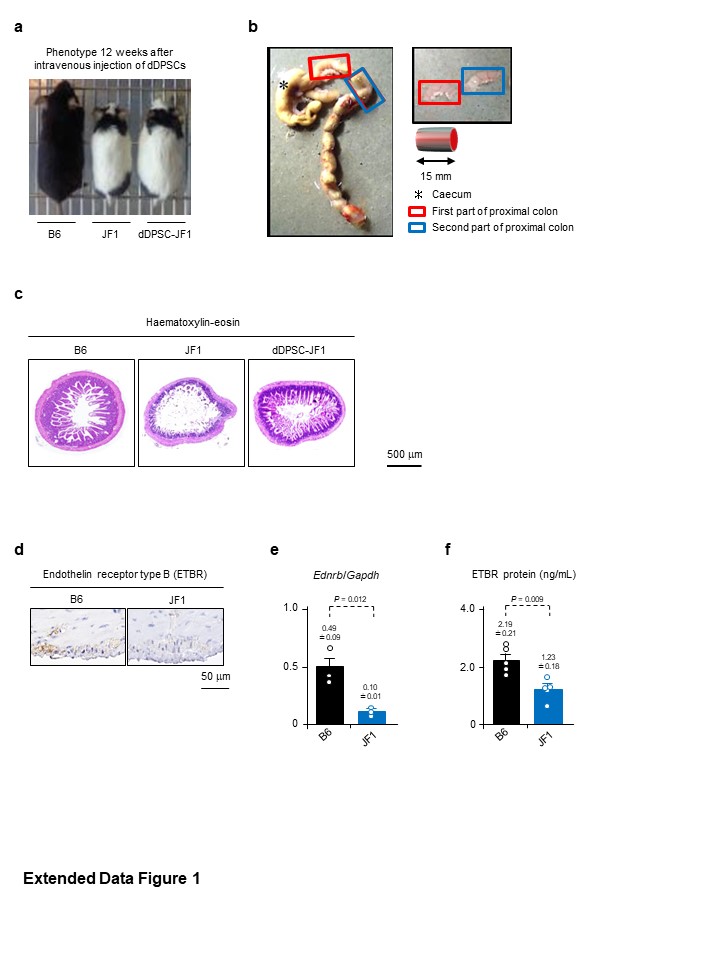


**Extended Data Fig. 1: The JF1 mouse with hypoganglionosis.**

**a**, Wild-type B6, JF1 and dDPSC-JF1 mice at P23W (12 weeks after dDPSC transplantation for dDPSC-JF1 mice). **b**, Stagnation of colonic contents in a JF1 mouse (×200). **c**, Haematoxylin-eosin-stained images of the jejunum (×400). Representative of ≥3 experiments. **d,** Cross-sections of the proximal colon immunostained for endothelin receptor type B (ETBR). **e**, *Ednrb* mRNA expression in the proximal colon (normalized to that of *Gapdh*) quantified by RT-qPCR (mean±SEM, *n*=3). **f,** ETBR protein expression in the proximal colon quantified by ELISA (mean±SEM, *n*=5). **a-d:** Representative of ≥2 independent experiments.


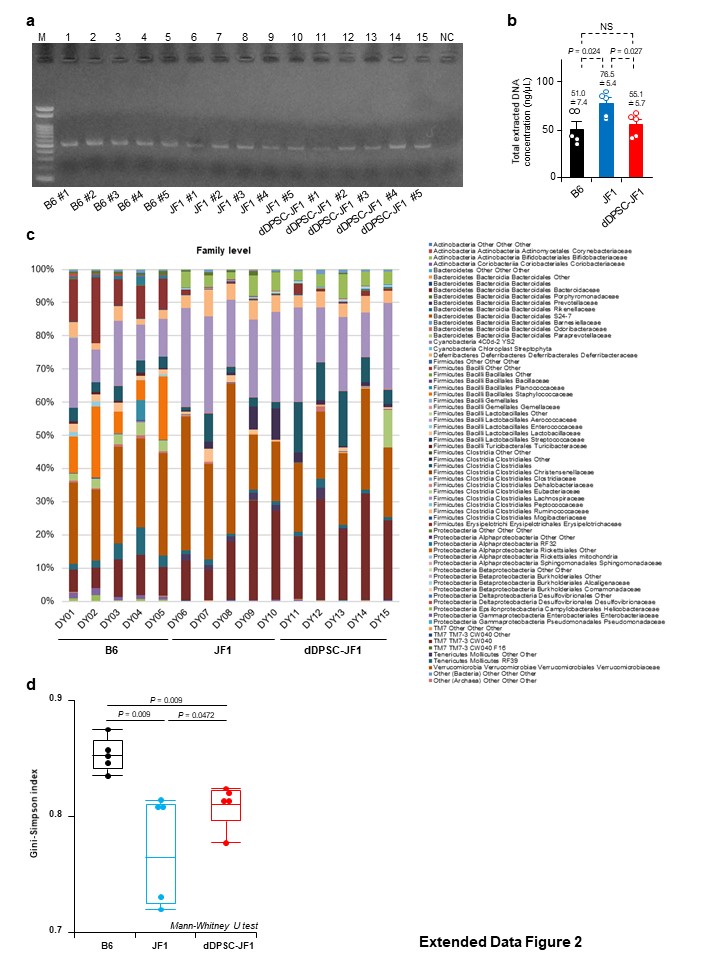


**Extended Data Fig. 2: Intestinal bacterial flora.**

**a**, The amounts of intestinal bacterial flora in B6, JF1 and dDPSC-JF1 mice (P23W) determined by RT-qPCR. **b**, Averaged data for the total amount of bacterial flora (mean±SEM, *n*=5). Center line, median; box limits, upper and lower quartiles; whiskers, extreme values. NS: not significant. **c**, Image summarizing the proportions of various bacteria (at the family level) in each individual animal. **d**, Gini-Simpson index determined as the reciprocal of Simpson’s index (1/D) (mean±SEM, *n*=5).


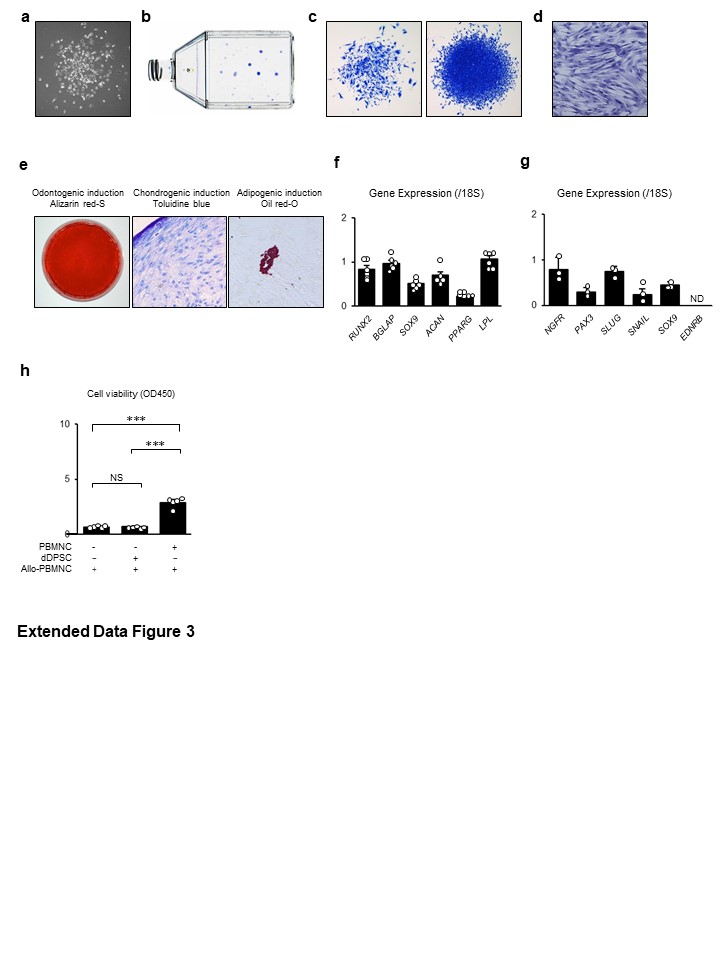


**Extended Data Fig. 3: Characteristics of dDPSCs.**

**a**, Colony forming unit-fibroblasts (CFU-Fs). **b**, Toluidine blue-stained colonies. **c**, Low-density (left) and high-density (right) CFU-Fs. **d**, Toluidine blue-stained dDPSCs (P3). **e**, Multipotent differentiation capacity of P3 dDPSCs. Images in **a**–**e:** Representative of ≥2 independent experiments.. **f**, Osteoblast-specific (*RUNX2*, *BGLAP*), chondrocyte-specific (*SOX9*, *ACAN*), and adipocyte-specific (*PPARG*, *LPL*) gene expression after appropriate induction (mean±SEM, *n*=3). **g**, Expressions of neural crest markers in P3 dDPSCs (mean±SEM, *n*=3). ND: not detected. **h**, Cell viability following mixed lymphocyte culture of dDPSCs or PBMNCs with gamma-irradiated allogenic PBMNCs (allo-PBMNCs; mean±SEM, *n*=3). ****P*<0.005; NS: not significant.


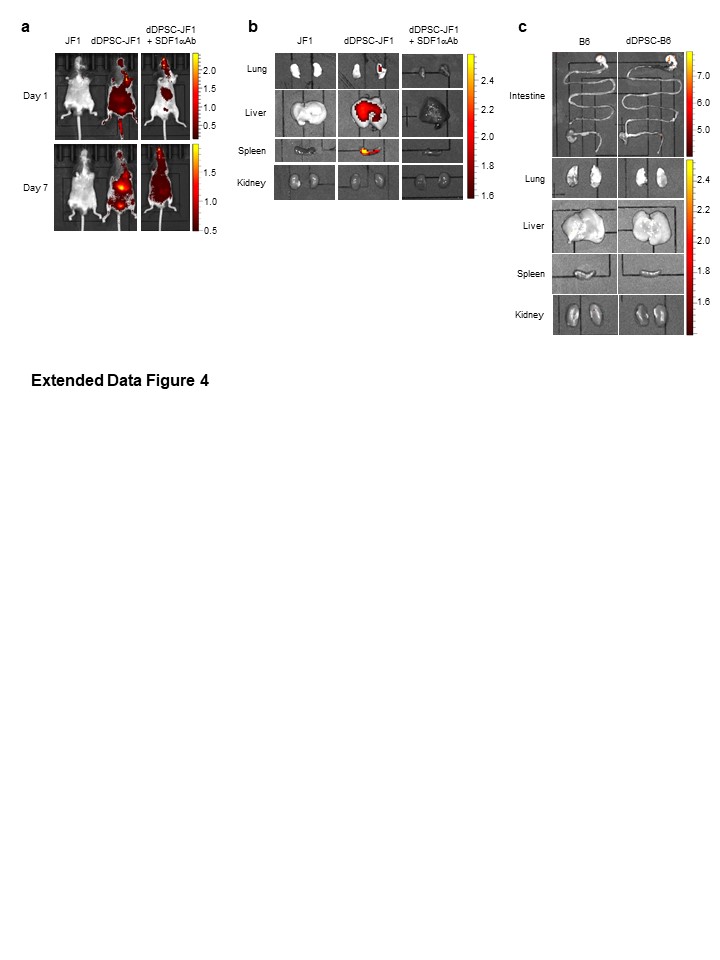


**Extended Data Fig. 4: Migration of transplanted dDPSCs.**

**a**, *In vivo* whole-body fluorescence imaging of mice (dorsal position) performed 1 day and 7 days after intravenous injection of DiR-labelled dDPSCs into JF1 mice (P11W). Some mice were pre-treated with anti-SDF1α antibody (100 mg/mouse) to establish whether CXCR4 receptors were involved in the migration process. **b**, *Ex vivo* fluorescence imaging of organs other than the intestine. **c**, *Ex vivo* fluorescence imaging of organs in B6 mice and DiR-dDPSC-transplanted B6 mice (dDPSC-B6). **a-c:** Representative of ≥2 independent experiments.


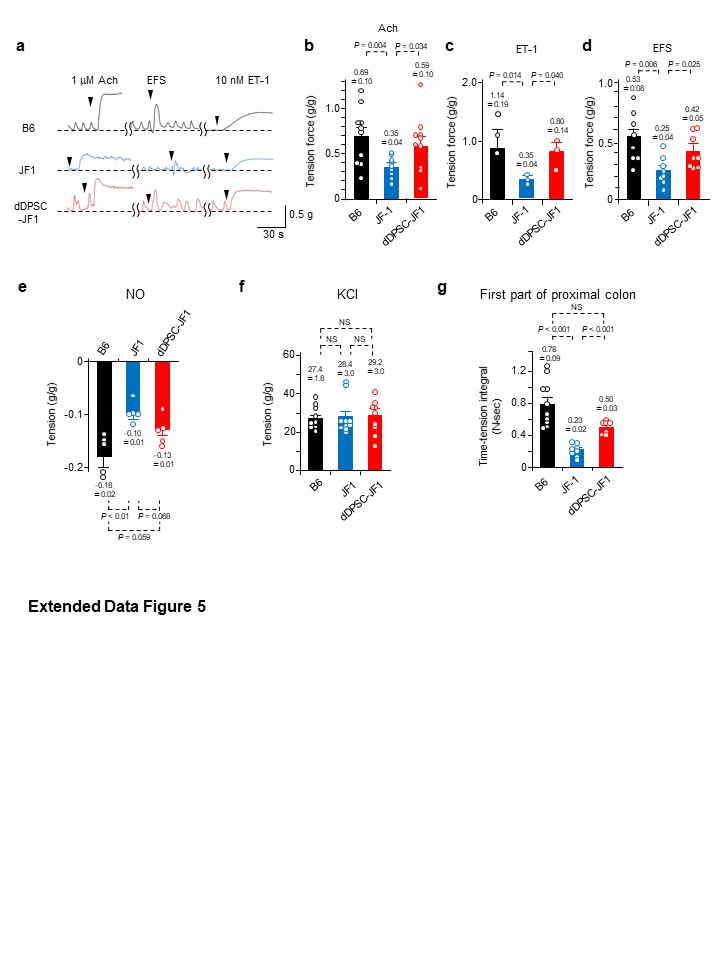


**Extended Data Fig. 5: dDPSCs improve colonic function.**

**a**, Traces showing the mechanical responses of the second part of the proximal colon to 1 µM Ach, 10 nM ET-1 and EFS (0.5 ms, 5 Hz, 50 V). Representative of ≥2 independent experiments. **b–f**, Mechanical responses of the second part of the proximal colon to 1 µM Ach (mean±SEM, *n*=10), 10 nM ET-1 (mean±SEM, *n*=3), EFS (mean±SEM, *n*=8), 10 µM NO (mean±SEM, *n*=6) and 60 mM KCl (mean±SEM, *n*=10). NS, not significant. **g**, Spontaneous activity in the first part of the proximal colon assessed as the area under the time-tension curve (mean±SEM, *n*=10).


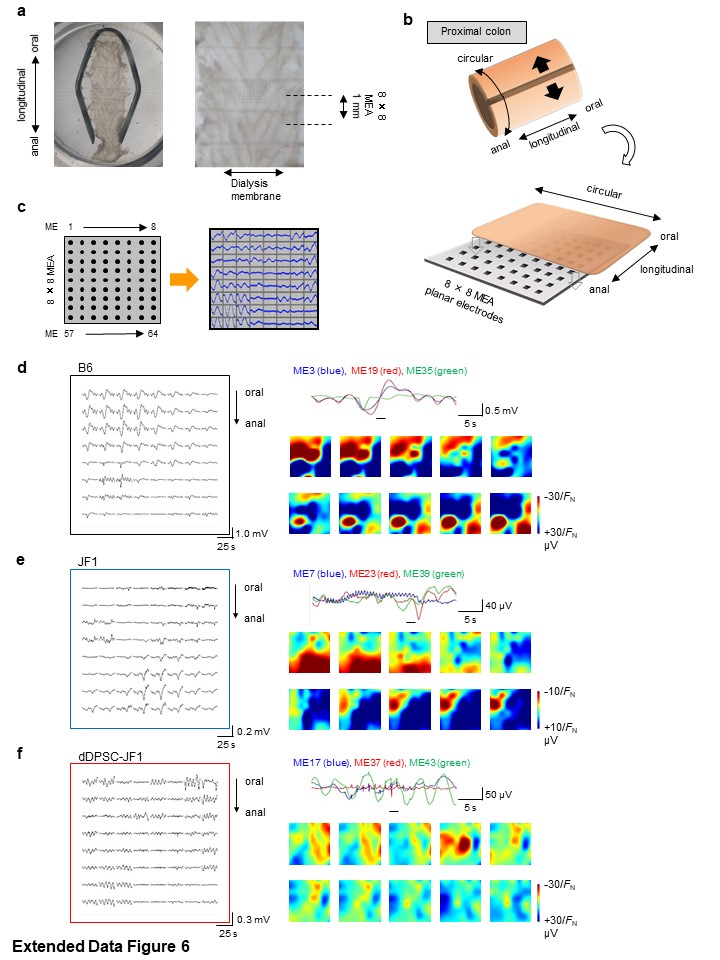


**Extended Data Fig. 6: Recording of electrical potentials.**

**a**, Recording of electrical potentials from a sample of proximal colon muscle using an 8×8 MEA. **b,** Procedures used for sample preparation. The proximal region of the colon was cut along the mesentery, and the mucosa was removed. The enteric muscle sample was mounted (longitudinal muscle layer facing downward) on an 8×8 MEA (interpolar distance = 150 µm). **c,** Positions of the 64 microelectrodes (MEs). **d–f,** Recordings of spontaneous electrical potentials in proximal colon samples from B6, JF1 and dDPSC-JF1 mice. **d-f:** Representative of ≥2 independent experiments.


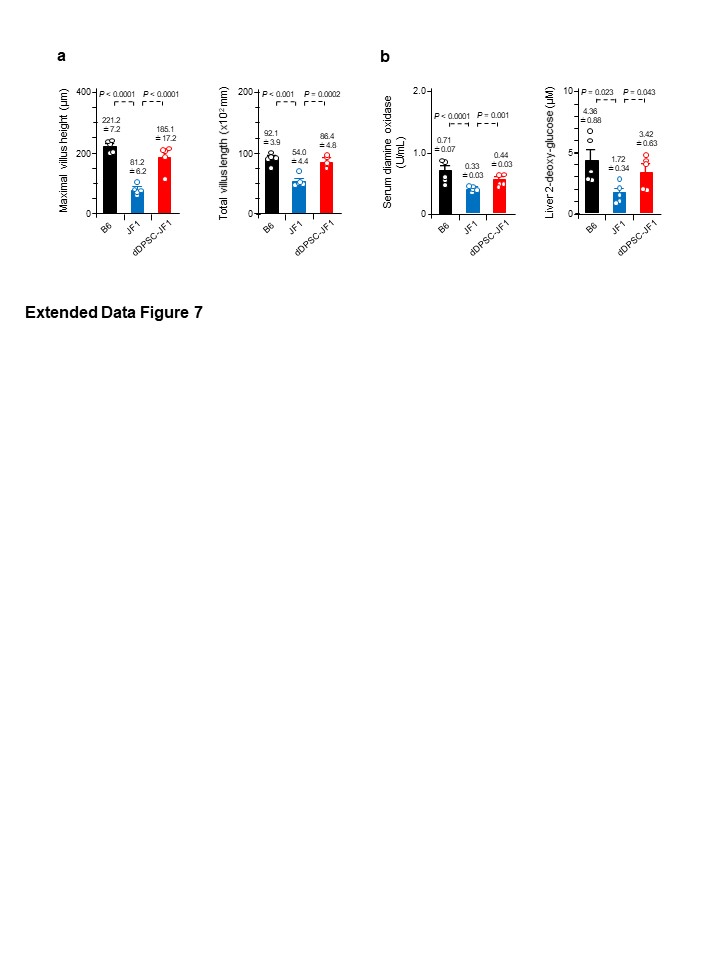


**Extended Data Fig. 7: Mucosal state and nutritional status.**

**a**, Maximal height and total length of the mucosal villi (mean±SEM, *n*=5). **b**, Serum diamine oxidase activity and hepatic 2-deoxy-glucose content (mean±SEM, *n*=5). All mice were used at P23W.


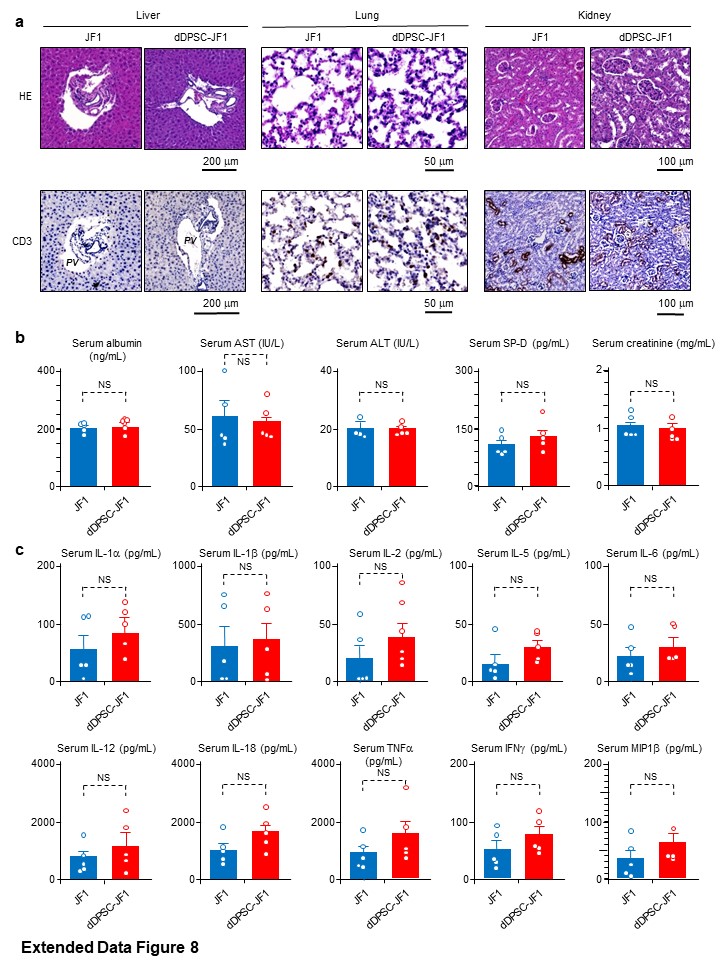


**Extended Data Fig. 8: Evaluation of off-target effects.**

**a**, Sections of the liver, lung and kidney stained with haematoxylin-eosin and anti-CD3 antibody. Magnification, ×100 (liver and kidney) and ×200 (lung). **a-c:** Representative of ≥2 independent experiments. **b**, Quantification of serum enzymes and metabolites associated with liver, lung and kidney damage (ELISA and colorimetric assays; mean±SEM, *n*=5). ALB, albumin; ALT, alanine aminotransferase; AST, aspartate transaminase; SP-D, surfactant protein D. **c**, Inflammation-related cytokines in the serum quantified by multiplex immunoassay (mean±SEM, *n*=5). IFNγ, interferon-gamma; IL, interleukin; MIP1β, macrophage infiltrating protein 1-beta; TNFα, tumour necrosis factor-alpha.
